# LC-MS/MS Identification of a Bromelain Peptide Biomarker from *Ananas comosus *Merr

**DOI:** 10.1155/2012/548486

**Published:** 2012-10-02

**Authors:** Eric R. Secor, Steven M. Szczepanek, Anurag Singh, Linda Guernsey, Prabitha Natarajan, Karim Rezaul, David K. Han, Roger S. Thrall, Lawrence K. Silbart

**Affiliations:** ^1^Department of Immunology, UConn Health Center, Farmington, CT 06030-1319, USA; ^2^The Carole and Ray Neag Comprehensive Cancer Center, UConn Health Center, Farmington, CT 06030-1628, USA; ^3^Allergy Group, Nestlé Research Center, P.O. Box 44, 1000 Lausanne, Switzerland; ^4^Center for Vascular Biology, UConn Health Center, Farmington, CT 06030, USA; ^5^Department of Allied Health Sciences, University of Connecticut, Storrs, CT 06269-2101, USA

## Abstract

Bromelain (Br) is a cysteine peptidase (GenBank AEH26024.1) from pineapple, with over 40 years of clinical use. The constituents mediating its anti-inflammatory activity are not thoroughly characterized and no peptide biomarker exists. Our objective is to characterize Br raw material and identify peptides in the plasma of Br treated mice. After SDS-PAGE in-gel digestion, Br (VN#3507; Middletown, CT, USA) peptides were analyzed via LC/MS/MS using 95% protein probability, 95% peptide probability, and a minimum peptide number = 5. Br spiked mouse plasma (1 ug/ul) and plasma from i.p. treated mice (12 mg/kg) were assessed using SRM. In Br raw material, we identified seven proteins: four proteases, one jacalin-like lectin, and two protease inhibitors. In Br spiked mouse plasma, six proteins (ananain, bromelain inhibitor, cysteine proteinase AN11, FB1035 precursor, FBSB precursor, and jacalin-like lectin) were identified. Using LC/MS/MS, we identified the unique peptide, DYGAVNEVK, derived from FB1035, in the plasma of i.p. Br treated mice. The spectral count of this peptide peaked at 6 hrs and was undetectable by 24 hrs. In this study, a novel Br peptide was identified in the plasma of treated mice for the first time. This Br peptide could serve as a biomarker to standardize the therapeutic dose and maximize clinical utility.

## 1. Introduction 

 Over 50% of Americans use some form of integrative and functional medicine with 20% using nutritional supplements or specific botanical formulations to complement their health care needs [1]. Concerns relating to the safety and efficacy of botanicals have increased due to the myriad of acute and chronic inflammatory conditions they are used for, either in isolation, or as in most cases, as adjuncts to pharmacological treatments [2–5]. As development of new drugs from botanical sources escalates, these products require confirmation of quality and characterization with sensitive and rigorous methods such as proteomics and mass spectrometry [6–9]. Also, the identification of biomarkers in clinically relevant animal models is critical to both inform and safely advance opportunities for human treatment [10–17].

Stem bromelain (Br) is an extract of the common pineapple *Ananas comosus (L.) *Merr. and is widely utilized as an anti-inflammatory compound due to its endogenous protease activity [18]. Br is categorized as an endopeptidase belonging to the Peptidase C1A subfamily (MEROPS database nomenclature; http://merops.sanger.ac.uk/) and may refer to a mixture of cysteine proteinases, peroxidases, acid phosphatases, glycosidases, and inhibitory proteins found throughout the pineapple fruit (EC 3.4.22.33) and stem (EC 3.4.22.32) [19, 20]. Historically, Br was identified as crude heterogeneous protein (2–5 proteolytically active components) with molecular mass found to vary between 8 and 28.5 kDa [21–23]. Although the nomenclature for Br is inconsistent, studies which utilized mass spectrometry have determined that Br has at least 8 proteolytically active components. These include stem Br (F4/F5), fruit Br, ananain (F9) comosain, SBA/a, and SBA/b [24–29]. 

The degree to which Br and its components are absorbed and retain function still remains to be fully elucidated. In experimental models of irritable bowel disease, it has been reported that the majority of orally administered Br remains undegraded and retains its enzymatic activity in the intestinal lumen of mice [30]. When administered orally, crude Br has been identified in human plasma via quantification with an immunoassay which detects Br antibodies [31]. Several studies confirm clinical effects of orally administered Br in cancer [32], rheumatoid arthritis [33], and osteoarthritis [34]; however, the characterization and detection of the individual Br peptides has not been reported. In addition, analytical techniques such as proteomics and LC-MS/MS have not been routinely applied to evaluate potential *in vivo* biomarkers for this botanical drug [35–37].

 The purpose of this study was to validate the components of a clinically used stem Br product, using MS and to determine if a Br peptide biomarker could be identified in the plasma of Br treated mice using LC-MS/MS. This is one of the first studies to report the components within a clinically used Br product and to identify a Br peptide (DYGAVNEVK) in plasma. This approach can now be applied to improve the characterization and quality control of Br in the marketplace. This biomarker will provide an important tool for standardizing the effective dose of the product in order to correlate the absorbed dose with therapeutic effects attributed to this complex product. 

## 2. Methods

### 2.1. Bromelain

Br (EC 3.4.22.32; catalog VNBR, lot number 3507) was obtained from RHG & Company Inc., Vital Nutrients (Middletown, CT, USA) and stored at 4°C in opaque containers. The identity of Br, extracted from *Ananas comosus *Merr. (common pineapple), was confirmed by matching its profile with the industry (Sigma) standards (via HPLC) as previously described [38]. Br was also independently tested for authenticity, potency, microbial contamination, residual solvents, heavy metals, and aflatoxins (Venture Laboratories, Lexington, KY, USA Eastern Analytical Laboratory, Old Saybrook, CT, USA Pharmline, Florida, NY, USA Covance, Madison, WI and Chemical Solutions Ltd., Mechanicsburg, PA, USA). 

### 2.2. Animals

Female C57BL/6J mice, 3 to 6 months of age and weighing 17 to 20 g, were purchased from the Jackson Laboratory (Bar Harbor, ME, USA) and housed conventionally in plastic cages with corncob bedding. The mouse room was maintained at 22 to 24°C with a daily light/dark cycle (light from 0600 to 1800 hours). Chow and water were supplied *ad libitum*. All mouse procedures were approved by the Animal Care Committee at the University of Connecticut Health Center (protocols 2009-508 and 2010-634).

### 2.3. Bromelain Treatment

 Individual stock solutions of stem Br were prepared. For *ex vivo* spiked serum experiments 100 mg Br was suspended in 20 ml diH_2_0; 10 *μ*L naïve mouse serum was spiked with 10 *μ*g Br. For i.p. treatment, 60 mg of Br was suspended in 125 mL of physiologic saline as previously described [39, 40]. Animals received one i.p. injection of Br (12 mg/kg) in 0.5 ml of physiological saline and serum collected at sacrifice over 24 hr time course. Serum samples were concentrated in 50 K Microcon Ultracel YM-50 (Millipore, Billerica, MA, USA) prior to SRM prep. 

### 2.4. In-Gel Digestion

 Br was processed with a modified RIPA buffer to concentrate plant associated protein and remove plant associated detritus and 1-dimensional SDS-PAGE separation was performed on the extract plant protein. Br samples were loaded into a 10% reducing mini-SDS-PAGE and separated via electrophoresis for 20 min. The gel lane corresponding to Br was then cut into 13 sections and subjected to proteolytic digestion on a ProGest workstation (Genomic Solutions) via reduction with DTT at 60°C, followed by a cool down to room temperature. Samples were then alkylated with iodoacetamide and digested in trypsin (12.5 *μ*g/mL) at 37°C for 4 h. Formic acid (5%) was added to stop the reaction and the supernatant was used for subsequent analyses.

### 2.5. LC-MS/MS

 Samples were analyzed using nano-ESI with a ThermoFisher LTQ Orbitrap XL LC/MS/MS. 30 *μ*L of trypsinized protein supernatant was loaded onto a 5 mm, 75 *μ*m ID C12 vented column (Jupiter Proteo, Phenomenex) at a flow rate of 10 *μ*L/min. A 30 min gradient elution was conducted over a 15 cm, 75 *μ*m ID C12 column at 300 nL/min. For identification of proteins in Br the mass spectrometer was operated in data-dependent mode in which the six most abundant ions were selected for MS/MS. The Orbitrap MS scan was performed at 60,000 FWHM resolutions as previously described [41, 42]. 

### 2.6. Mass Spectrometry Data Analysis

MS/MS data were first searched using Mascot (http://www.matrixscience.com/) with the following parameters: type of search MS/MS ion search, taxonomy-all kingdoms, enzyme-trypsin, fixed modifications-carbamidomethyl (C), variable modifications-oxidation (M), acetyl (N-term), pyro-glu (N-term Q), deamidation (N,Q), mass values-monoisotopic, protein mass-unrestricted, peptide mass tolerance- ± 10 ppm, fragment mass tolerance- ± 0.5 Da, and max missed cleavages-2. Samples were further analyzed using the Scaffold algorithm (http://www.proteomesoftware.com/) with the DAT files generated by Mascot. Positive identification was determined if a minimum of 5 peptides matched per protein with minimum probabilities of 95% at the protein level and 95% at the corresponding peptide level.

### 2.7. Fractionation of Serum Proteins

 In a first step, 10 *μ*L of mouse serum was diluted by the addition of 490 *μ*L of PBS and serum proteins were separated by ultrafiltration using a centrifugal concentrator Centricon YM-50 to obtain a protein fraction of <50 kDa. The filtrates were concentrated by TCA precipitation using 72.0 *μ*L of 100% TCA and 15.0 *μ*L of 10% cholic acid/1 mL filtrate. The precipitated pellets were washed three times with 1 mL of acetone. The pellet was briefly dried at room temperature, and then dissolved in 30.0 *μ*L of 0.05 M NaOH supplemented with 5.0 *μ*L of 6X SDS-PAGE sample buffer and boiled for 5 min. 

### 2.8. Serum Sample Preparation and LC-MS/MS Analysis

 Each sample was separated on a 10% NuPage gel (Invitrogen).Silver staining was used to detect proteins after electrophoretic separation on polyacrylamide gels. As no prominent protein bands were detectable in area of interest, three gel slices from each lane ranging from 23 to 39 kDa areas were excised and in-gel trypsin digestion was performed as described previously (1, 2). Samples were then resuspended in buffer A (5% ACN, 0.4% acetic acid, 0.005% heptafluorobutyric (HFBA) acid in water) and stored at −20°C until further analysis.

The peptide digests were sequenced using a high throughput tandem mass spectrometer, LTQ ESI ion trap (ThermoFinnigan, Palo Alto, CA, USA) equipped with a commercial nanoelectrospray device as described previously (1, 2). Each sample was directly loaded onto a 10 cm × 100 *μ*m capillary column packed in-house (Magic C18; Michrom BioResources, Auburn, CA, USA) by means of a micro-autosampler (Famos, Dionex, Sunnyvale, CA, USA). The column was previously equilibrated with solvent A (5% acetonitrile, 0.4% acetic acid, and 0.005% HFBA). Peptides were eluted with a linear gradient from 100% solvent A to 80% solvent B (95% acetonitrile, 0.4% acetic acid, and 0.005% HFBA) in 85 min at an elution rate of 200 nL/min. A Hewlett Packard 1100 solvent delivery system with flow splitting (Hewlett Packard, Palo Alto, CA, USA) was used. Peptides were eluted directly into the LTQ ESI ion trap mass spectrometer equipped with data-dependent acquisition, and a scan was performed. Each full MS scan was followed by one MS/MS scan of the most intense peaks in the full MS spectrum with dynamic exclusion enabled to allow detection of low abundant peptide ions. Auto sampler loading, mass spectrometric scan functions, and HPLC solvent gradients were controlled by the Xcalibur data system (ThermoFinnigan, Palo Alto, CA, USA).

### 2.9. Analysis of Serum MS/MS Data

 All LC-MS/MS runs were processed in the following way as described previously [43, 44]. All the mass spectrometry raw files were converted to  .dat files using Xcalibur software (version 1.4 SR1). Peak lists were automatically extracted using the extract  .ms program with default parameters, except that filtering was turned off. All the  .dat files were searched against a local copy of the nonredundant pineapple (*Ananas comosus*) database from the NCBI, National Institutes of Health, and Advanced Biomedical Computing Center using the SEQUEST algorithm (SEQUEST-PVM version 27 (revision 0)) and SEQUEST parameters were as follows: all the filtering thresholds were off; mass tolerance of 1.5 Da for precursor ions and 0.5 Da for fragment ions; full tryptic constraint allowing one missed cleavage; allowing oxidization (+16 Da) of methionine. The database search results were processed using the INTERACT program and filtered with the following criteria: Xcorr cutoff values of 1.8, 1.8, and 3.1 for 1+, 2+, and 3+ peptides, respectively, ΔCn ≥ 0.1. Finally, all potential MS/MS spectral matches were subjected to manual inspection as a final validation step prior to acceptance as a valid peptide identification. The spectral count of identified peptides from three runs of each sample was used for semiquantitative measurement of bromelain-mediated events.

## 3. Results 

### 3.1. Bromelain Identity and Quality Control

In the present study, the Br raw material was obtained from RHG & Company Inc., Vital Nutrients imported from Thailand. Prior to manufacturing, Br was independently tested for identification and quality. A summary of results is provided in Table 1. Identification of Br was confirmed via HPLC with an industry (Sigma) standard. The proteolytic activity (a measure of potency) of Br was 2931 GDU/gm. All residual solvents residues (from a total of 51 solvents and 166 chemical analytes) tested were within allowable USP Limits. Raw material was negative for *E. coli*, *Salmonella, S. aureus,* and *P. aeruginosa* and below limits for mold yeast (160 CFU/gm; allowable ≤300/gram) and Enterobacteriaceae (≤10/gram; allowable ≤10/gram). Aflatoxins (B1, B2, G1, and G2) were negative and all heavy metals tested (arsenic, lead, cadmium, and mercury) were below allowable limits. 

### 3.2. Characterization of Bromelain Associated Proteins by Peptide Sequencing 

**Figure 1:**
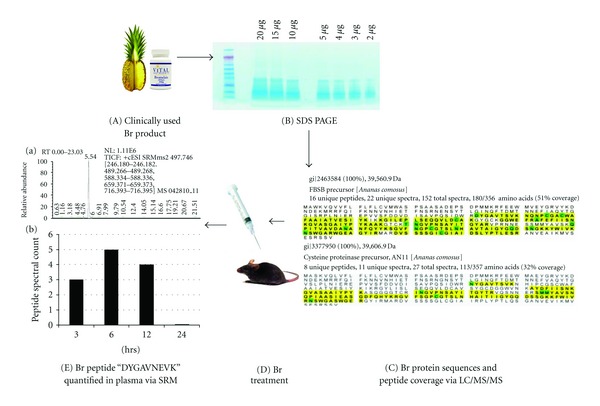
Bromelain characterization flow diagram. A clinically used quality verified Br product (A) was processed and electrophoresed on 1D SDS PAGE mini gel (B). The mobility region was excised and gel digests were analyzed via LC-MS/MS for protein identification. Production data were searched against all kingdoms of the NCBInr database via Mascot search engine and files were parsed into the Scaffold proteome program (C). After i.p. Br treatment (D) plasma samples were assed via SRM for presence of Br specific peptides and DYGAVNEVK was identified and quantified over 24 hrs (E).

The overall work flow is represented in Figure 1. A clinically used Br product was chosen for analysis (Figure 1(A)). After quality control analysis was completed, mass spectrometry on raw material was initiated. Post SDS-PAGE separation (Figure 1(B)) in the region of the gel corresponding to 8–28 kDa was removed and processed with trypsin digestion. The resulting peptides were analyzed by LC/MS/MS (Figure 1(C)), data were processed in the Scaffold proteome program after which *in vivo* Br treatment initiated (Figure 1(D)), and serum was analyzed (Figure 1(E)). By setting the data filtering limits (protein probability ≥95%, peptide probability ≥95%, minimum peptide number = 5),  we identified six proteins in Br raw material (Table 2) with 100% probability of match. Four were proteases-FBSB precursor, FB1035 precursor, cysteine proteinase precursor ANll, and Ananain. One was a Br inhibitor and one a jacalin-like lectin. All proteins were identified in Br Raw material as well as spiked mouse plasma. 

**Figure 2:**
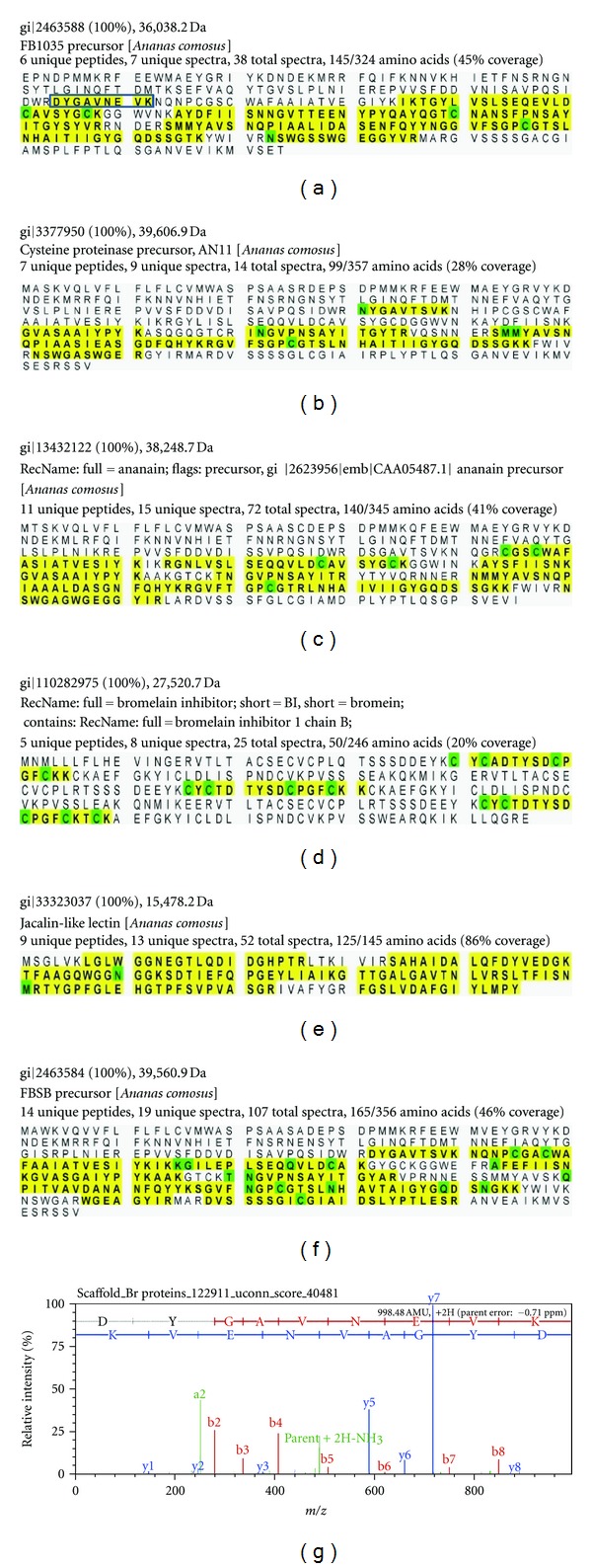
Br protein quantitation. Data filtering limits were set at a protein probability ≥95%, peptide probability ≥95%, and a minimum peptide number of 5. Six proteins were quantitated with a 100% probability of match. FB1035 precursor (a), Cysteine proteinase precursor ANll (b), Ananain (c), Br inhibitor (d) FBSB jacalin-like lectin (e), and the FBSB precursor (f). The ion spectrum (g) is shown for the DYGAVNEVK peptide in the FB1035 precursor (box in A).

The percentage of amino acid peptide coverage for each protein (Figures 2(a)–2(f)) is depicted in yellow and sites of predicted amino acid posttranslational modification were in green. The ion spectrum (Figure 2(g)) is also shown for the peptide “DYGAVNEVK” in the FBSB precursor protein. The DYGAVNEVK peptide had a probability of 100% and a Mascot ion score of 60.

### 3.3. Identification of Br Peptide Biomarker from Mouse Plasma after Br Treatment

**Figure 3:**
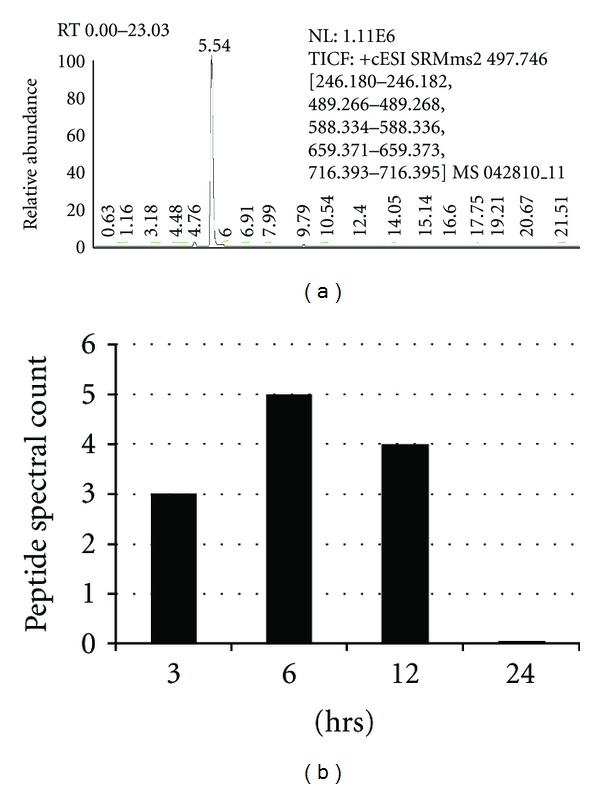
Bromelain LC-MS/MS and DYGAVNEVK peptide quantification. Individual mice were treated with Br (one–12 mg/kg i.p. injection) and peripheral blood was collected via cardiac puncture at 3, 6, 12, and 24 hrs and processed. Plasma was collected and processed, and filtrate was analyzed by LC-MS/MS. The total number of spectra of peptide “DYGAVNEVK” from Br protein FB1035 precursor was identified in sera peaking at 6 hrs over the 24 hr time course.

The identification of Br specific peptides in Br raw material and *ex vivo* spiked serum (assessment of matrix effects) were critical in standardization of LC-MS/MS for the next phase of experiments, identification, and quantification of a Br peptide in the plasma of mice following Br treatment. Plasma was collected and processed, and the filtrate was analyzed by LC-MS/MS. For the analysis, the most abundant peak in the chromatograph eluded at 5.54 min (Figure 3(a)) and this peptide ion spectrum was consistent with DYGAVNEVK. The spectral count of peptide “DYGAVNEVK” from Br was quantified in sera over the 24 hr time course, peaking at 6 hr (Figure 3(b)).

## 4. Discussion

According to the Natural Foods Merchandiser, total natural products industry sales reached a record $81 billion in 2010 [45]. This widespread consumption of supplements and botanicals often ignores the issue of safety, consistency, and efficacy, which are often lacking despite longstanding traditional uses for select products [46, 47]. Br is a proteolytically active pineapple extract with decades of over-the-counter use as anti-inflammatory and digestive aid [18, 21, 32, 39, 48, 49]. Although accessible in numerous product combinations (with other enzymes, antioxidants, vitamins, minerals, or botanicals), Br has not been thoroughly characterized and no biomarker exists to evaluate its absorption or the therapeutic properties of individual components of the product. Our objective was to characterize a commercially available Br product via LC/MS/MS and identify a peptide via SRM in the plasma of Br treated mice. In this study, Br peptides were characterized and a marker peptide, DYGAVNEVK, was quantified in the plasma of Br treated mice. This Br peptide may serve as a useful biomarker to help standardize therapeutic dosage and maximize its clinical utility.

### 4.1. Br Identification and Assessment of Quality

Currently manufacturers who produce Br supplements are expected to provide specific details about their ingredients on the label [46, 47]. This includes the amount (mg or gm) delivered per capsule, the broad nonspecific protein digesting activity in gelatin digesting units (GDU) or milk clotting units (MCU), and the presence of other active or nonactive ingredients such as excipients, binders, or fillers. In addition, manufacturers should provide a certificate of analysis which identifies the plant part utilized, extraction method, and verification of the validity of botanical extract. With the anticipated enforcement of the DSHEA [50], detailed results of quality control testing (solvent residues, microbial contaminants, heavy metals, and pesticide residues) will also be required [51, 52]. Without this information, the manufacturer, physician, and consumer has no detailed knowledge of the individual constituents being delivered within Br supplements, nor details of optimal product composition and variation [52–54]. Moreover, once products such as Br are ingested, there is no *in vivo* mechanism (data or bio markers) for monitoring the absorbed dose or therapeutic effectiveness of the product. 

In order to characterize Br being utilized by consumers, we chose a commercially available product distributed to physicians for use in our research. Although there were several industrial sources available, most are “for research purposes only” such as Sigma's and may not represent the Br manufactured for medicinal use. Also, handling, testing and consistency of natural products may vary greatly. The Br we utilized was independently tested by analytical laboratories for enzymatic activity and contamination from solvents, microbials, aflatoxins, and heavy metals (Table 1). Organoleptic techniques, which are still used to identify pineapple and its mature stem, are combined with HPLC to confirm the Br extract. Although Br is composed of both cysteine proteases and inhibitory proteins, only the net enzymatic activity of Br (2931 DU/gm for our lot) is cited on product labels and the ratio and consistency of individual proteins is unknown. We are unaware of any other botanical studies which conduct this level of quality control testing on clinically used products.

### 4.2. Peptide Identification and Protein Verification via LC-MS/MS

Before analysis could be applied to the serum of i.p. Br treated mice, the peptides and protein(s) present in Br stem extract had to be identified and verified in order to choose suitable peptide(s) for use as biomarkers. Separation of Br raw material via LC and identification of Br peptides via MS/MS resulted in the identification of five protease (Figure 2): (1) FB1035 gi2463588, (2) cysteine proteinase AN11 gi3377950, (3) gi2623956, (4) FBSB precursor gi2463584, and (5) stem bromelain gi115139, a jacalin-like lectin gi33323037, and a Br inhibitor protein gi110282975 [55, 56]. Two Br protein sequences identified, VPQSIDWRDSGAVTSVKNQG in ananain and AVPQSIDWRDYGAVTSVKNQN in FBSB precursor, correspond and confirm previous investigations on the comparisons of these N-terminal amino acid sequences performed by Napper et al. [27]. In our initial analysis we did not identify the 2KDa cysteine proteinase, comosain which had been previously described [27, 57, 58]. However, adjusting the filter limits from a minimum peptide number of 5 down to 2 (protein probability ≥95%, peptide probability ≥95%) did confirm that in addition to comosain (gi685082, VPQSIDWRNYG AVTSVKNQG), the Br inhibitor IV H chain (gi159163781) as well as chitinase A (gi189095136) chitinase C (gi125995173), and cystatin (gi33323039), were all present in the Br raw material. 

In contrast to stem Br, which has been the subject of numerous basic and clinical studies, there are very few studies on individual Br proteins. A novel ananain comosain combination product, Vianain, has been formulated for topical wound treatment and debridement due to its inherent protease activity [59], and the highly purified bromelain protease F9 (or ananain) has been investigated for its ability to reduce CD44 mediated adhesion in lymphocytes [38]. Although the Br specific jacalin-like lectin has been previously identified (EMBL:AAQ07258.1), no specific literature exists which describes its distribution and potential function in *Ananas comosus*. Similar jacalin-like lectins have been identified in several plant families (Moraceae-jackfruit, Gramineae-Triticum aestivum, or wheat) and function in protein-carbohydrate recognition through cell surface binding of carbohydrates such as mannose [60]. Jacalin-like lectins may also have a role clinically as there is evidence in enhancing pathogen resistance and facilitate binding of human IgA [61, 62]. 

Although controversy exists regarding the extent to which Br is absorbed and present in the serum, Castel has identified the presence of anti-Br antibodies in human plasma after oral administration [31] while in separate studies White and Seifert noted that both orally and intraduodenally administered, ^125^I-labeled Br was absorbed and present in the serum of rats [63, 64]. However, there are no reports of unique Br peptide biomarkers present in plasma following exposure. In order to determine if Br peptides were present in serum, we utilized LC-MS/MS, a technique associated with the selective and sensitive detection of small molecules (<1000 Da) which is widely used in clinical, pharmaceutical, and environmental monitoring fields [65]. As part of the SRM assay process, mouse plasma samples were spiked with Br and processed using an in-solution digestion protocol that requires reducing and alkylating the sample before trypsin digestion and analysis to determine recovery and evaluate matrix affects. In Br spiked mouse plasma, six proteins (FB1035 precursor, cysteine proteinase AN11, ananain, bromelain inhibitor, the jacalin-like lectin, and FBSB precursor) were identified and correspond to Br raw material (Table 2). 

The unique peptides DYGAVNEVK, TGYLVSLSEQEVLDCAVSYGCK, and NSWGSSWGEGGYVR within the FB1035 gi*|*2463588 protein were previously observed in an LC-MS/MS analysis of bromelain extract. When plasma from Br treated mice was evaluated, we identified the DYGAVNEVK biomarker peptide. The total number of spectra identified or spectral count for DYGAVNEVK was found to peak at 6 hrs over the 24 hr time course evaluated after initial intraperitoneal Br treatment. Although the specific activity of this peptide is unknown, DYGAVNEVK is present in one of the four proteolytically active proteins (FB1035) within Br which contribute to its therapeutic effects. Future studies may determine the contribution of individual peptides and set the stage for their use as compounds to standardize Br beyond its peptidase activity, which is becoming the gold standard within the botanical extract industry [66–68]. By utilizing standardized well-established analytic methods such as LC-MS/MS, we hope to develop a robust, high throughput assay which has the sensitivity to determine if protein or peptide ratios vary between distinct Br products and if this affects their enzymatic activity. In addition, as Br products are widely used for numerous inflammatory and immunological conditions, it is critical to quantify Br peptides for use as potential biomarkers in human samples as well. This information will provide data for both safety and efficacy studies as well as optimizing the therapeutic dose needed for clinical utility.

## Figures and Tables

**Table 1:** Bromelain identity and quality control data.

Item profile	Specification		Result	Method
Botanical-pineapple	*Ananas* sp.		*Ananas comosus*	Visual
Plant parts used	Mature plant stem		Mature plant stem	Visual
Botanical extract	Bromelain		Bromelain	Maltodextrin diluent
Identification	Standard Match (Sigma)		Conforms to standard	HPLC*
Activity, potency	≥2400 GDU**/gram		2931 GDU/gram	GDU Assay
Solvent Residues	LOQ^‡^ Limits USP			
Methanol	1000 ppm	3000 ppm	<1000 ppm	Gas chromatography
Ethanol	1000 ppm	5000 ppm	<1000 ppm	Gas chromatography
Diethyl ether	2 ppm	5000 ppm	<2.0 ppm	Gas chromatography
Acetone	50 ppm	5000 ppm	<50 ppm	Gas chromatography
2-Propanol	200 ppm	5000 ppm	<1200 ppm	Gas chromatography
Dichloromethane	2 ppm	600 ppm	<2.0 ppm	Gas chromatography
n-Hexane (C6)	2 ppm	290 ppm	<2.0 ppm	Gas chromatography
Ethyl acetate	20 ppm	5000 ppm	<20.0 ppm	Gas chromatography
Xylenes (0, M, P, EB)	1 ppm	2170 ppm	≤1.0 ppm	Gas chromatography
Microbial Profile	(CFU/gm)		(CFU/gm)	
Total mold and yeast	≤300/gram		160	MLP° USP 27
*E. coli *	Negative		Negative	MLP USP 27
*Salmonella *	Negative		Negative	MLP USP 27
*Enterobacteriaceae *	≤10 CFU/gram		<10/gram	MLP USP 27
*S. aureus *	Negative		Negative	MLP USP 27
*P. aeruginosa *	Negative		Negative	MLP USP 27
Aflatoxin Profile (B1,B2,G1,G2)	Aflatoxins ≤20 ppb		Negative	HPLC
Heavy metal profile				
Arsenic	≤3 ppm		<0.50 ± 0.5 ppm	ICP-MS!
Lead	≤10 ppm		0.08 ± 0.05 ppm	ICP-MS
Cadmium	≤3 ppm		<0.25 ± 0.25 ppm	ICP-MS
Mercury	≤2 ppm		<0.10 ± 0.1 ppm	ICP-MS

*HPLC: high-performance liquid chromatography, **GDU's: gelatin dissolving units; LOQ^‡^: limit of quantification, °microbial limit tests; ! ICP-MS: inductively coupled plasma-mass spectrometry; ^#^CDFA: California Department of Food and Agriculture.

**Table 2:** Bromelain LC/MS/MS protein identification: raw material corresponds to spiked plasma.

Protein name	Accession number	MW (kDa)	Unique peptides	Match probabilty	Sequence aa coverage	ID in raw material and plasma
(A) FB1035 precursor	gi∣2463588	36.2	14	100%	165/356 (46%)	Y
(B) Cysteine proteinase precursor N11	gi∣3377950	39.6	7	100%	99/357 (28%)	Y
(C) Ananain	gi∣2623956	38.2	11	100%	140/345 (41%)	Y
(D) Bromelain inhibitor	gi∣110282975	27.5	5	100%	50/246 (20%)	Y
(E) Jacalin-like lectin	gi∣33323037	15.5	9	100%	125/145 (86%)	Y
(F) FBSB precursor	gi∣2463584	39.6	14	100%	165/356 (46%)	Y
